# Developmental Control of a Lepidopteran Pest *Spodoptera exigua* by Ingestion of Bacteria Expressing dsRNA of a Non-Midgut Gene

**DOI:** 10.1371/journal.pone.0006225

**Published:** 2009-07-13

**Authors:** Honggang Tian, Han Peng, Qiong Yao, Hongxin Chen, Qi Xie, Bin Tang, Wenqing Zhang

**Affiliations:** 1 State Key Laboratory of Biocontrol, School of Life Sciences, Sun Yat-sen University, Guangzhou, China; 2 Institute of Genetics and Developmental Biology, Chinese Academy of Sciences, Beijing, China; New Mexico State University, United States of America

## Abstract

**Background:**

RNA interference (RNAi) induced through double stranded RNA (dsRNA) has been used widely to study gene function in insects. Recently, it has been reported that gene knockdown in several insects can be induced successfully through feeding with dsRNA. However, it is still unknown whether phenotypic silencing of genes not expressed in the midgut occurs after ingestion of insect dsRNA.

**Principal Findings:**

Using chitin synthase gene A (*SeCHSA*) as the target gene, which is expressed in the cuticle and tracheae of the lepidopteran pest *Spodoptera exigua*, we showed that the growth and development of *S. exigua* larvae fed *Escherichia coli* expressing dsRNA of *SeCHSA* was disturbed, resulting in lethality. In the 4th and 5th larval instars, prepupae, and pupae, the mean survival rates of insects fed the dsRNA-containing diet were 88.64%, 74.24%, 68.43% and 62.63% respectively. The survival rates in the 5^th^ instar larvae, prepupae and pupae stages were significantly lower than those of all controls, and significant lethality differences were also found between dsSeCHSA treatment and dsControl or ddH_2_O control in the 4^th^ instar larvae. The effects of ingesting bacterially expressed dsRNA on transcription of the target gene, tissue structure, and survival rates of insects were dose-dependent.

**Conclusions:**

Our results suggest that *SeCHSA* dsRNA may be useful as a means of insect pest control.

## Introduction

RNA interference (RNAi) has emerged as a powerful tool for the rapid analysis of gene function in a variety of organisms. RNAi also shows great promise for use in biotechnology. Relevant applications include the capacity to avoid unwanted transgene silencing in genetically engineered lines and the exploitation of various types of silencing to inactivate undesirable genes [1], [2]. Direct microinjection is the most commonly used procedure for delivery of double-stranded RNA (dsRNA) into organisms. However, scientists in a variety of fields have been exploring more simple and convenient means of dsRNA delivery, including soaking [3], oral feeding [4], and transgenic plant expression [5]. Of these delivery methods, transgenic plants expressing viral dsRNAs, including *Arabidopsis thaliana* [6] and the sugar beet *Beta vulgaris* [5], have been used to control plant pathogens. The application of RNAi in insect pest control has lagged behind its application in plant pathogen control following years of unsuccessful attempts [2]. However, in 2007, two groups made major progress in the exploitation of transgenic plants engineered to express insect dsRNAs for entomological research and field control of insect pests [7], [8]. These exciting results encourage us to believe that transgene-encoded ingestible dsRNA plant may one day be used for insect pest control.

In 2006, the global area in use for biotech crops continued to climb for the tenth consecutive year at a sustained double-digit growth rate of 13%, reaching 102 million hectares. However, none of the transgenes employed in large-scale commercialized transgenic plants have insect origins. Recent results show that RNAi induced in insects after ingestion of plant-expressed hairpin RNA offers promise for insect pest control [2], [7], [8]. However, identification of a suitable gene for transgenic RNAi in plants is very important; such a gene should not only have insecticidal effects on the target pests, but should also be safe to the pests' natural enemies and to human beings. The achievement of these goals requires a method for large scale screening from pest gene pools. Fortunately, RNAi through dsRNA ingestion provides a good method for discovery of appropriate gene(s). The RNAi feeding protocol has several advantages over microinjection; most notably it is more convenient and less laborious [9]. In insects, Tuner and his colleagues first reported that RNAi can be triggered by oral delivery of dsRNAs for a gut gene *EposCXE1*, and an antennae gene *EposPBP1*, using a microvolume syringe to deliver dsRNA to the mouthparts of a lepidopteran *Epiphyas postvittana* [10], later, it was found that feeding of dsRNA to the cricket *Gryllus bimaculatus* [11], and termite *Reticulitermes flavipes* [12], can also trigger an RNAi response. In particular, gene silencing in *R. falvipes* had lethal effects. However, in other lepidopteran insects, it is still unknown whether the RNAi response and insecticide effects can be produced, especially for a non-midgut gene through voluntary ingestion of dsRNA.

dsRNAs for ingestion protocols can be synthesized using an RNAi kit and produced in bacteria. *Escherichia coli*-mediated delivery of dsRNA was reported in *Caenorhabditis elegans* in 1998 [13] and in planarians in 2003 [14]. In comparison with producing dsRNA *in vitro* with a kit, bacterially expressed dsRNA is more cheaply and is more easily used in large scale gene function analysis [15]. Therefore, we used ingestion of bacterially expressed dsRNA to study a non-midgut gene in a lepidopteran pest, *Spodoptera exigua*.

Insect chitin synthases are key enzymes for cuticle, trachea, and midgut development [16], [17]. They are encoded by two classes of genes: CHSA and CHSB. CHSA genes are specifically expressed in ectodermal cells, including epidermal and tracheal cells, while CHSB genes are restricted to gut epithelial cells that produce the peritrophic matrix of the midgut [18]. Since chitin is mainly found in arthropods and fungi but not in vertebrates and plants [19], chitin synthesis has been an ideal target for insect growth regulators [16], [18]. Chitin synthase gene A of the *S. exigua* (*SeCHSA*) is not expressed in the midgut, and our previous research using injection of *SeCHSA* dsRNA into *S. exigua* larvae demonstrated disruption of the larval development [20]. Thus, we used the chitin synthase gene A in this study.

The SID-1 (systemic RNA interference defective) gene has been suggested to act as a dsRNA signal channel in systemic RNAi in *C. elegans* [21]. To date, SID-1 genes have been found in almost all animal genomes with the exception of dipteran genomes; this may be one reason why feeding *Drosophila* yeast-expressed dsRNA failed as a means of inducing RNAi [22]. In the present study, we first characterized a partial sequence of a SID-1 like gene in *S. exigua* in order to confirm that this vital systemic RNAi molecule is present in this species. Subsequently, we fed bacterially expressed *SeCHSA* dsRNA to *S. exigua* larvae and seven days later we observed an RNAi response; larvae could not completely remove their old cuticles, exhibiting lethal phenotypes and insecticide effects. Further examination of the lethal phenotype showed that treated larvae exhibited decreased endocuticle and double-cuticles. Our results suggest RNAi can effectively inhibit gene expression in lepidopteran insects when insects are fed with dsRNA to non-midgut genes. Ingestion of bacterially expressed dsRNA may be applicable for large scale optimal gene screening for ingestible RNAi plants in insect pest control.

## Results

### Characterization and expression pattern analysis of *sid-1* like gene in *S. exigua*

**Figure 1:**
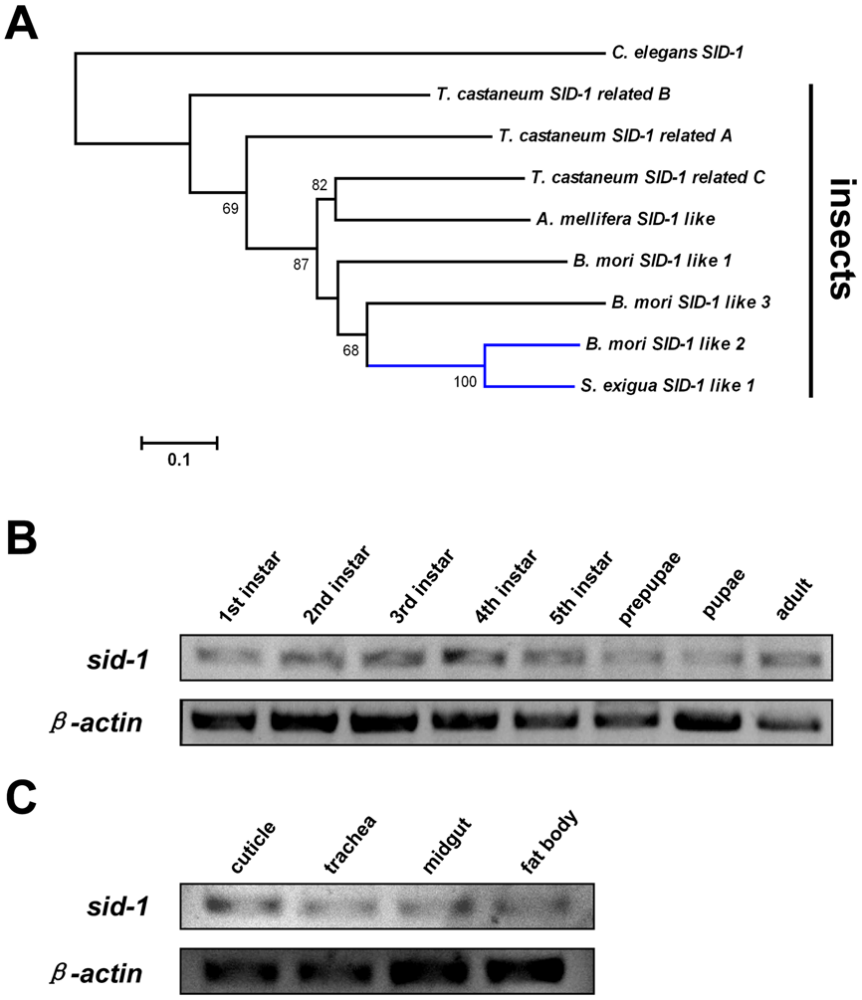
Phylogenetic relationship analysis and expression pattern of *sid-1* in *Spodoptera exigua*. (A) The phylogenetic analysis of deduced SID-1 amino acids of insects and nematode *C. elegans* were conducted using MEGA 4.0, nodes with distance bootstrap values of >50% were indicated, and the branches with blue color represents the closest group with *S. exigua* SID-1 like 1. (B) Developmental expression patterns of *sid-1* like 1 gene in *S. exigua* from 1^st^ instar larvae to adult. (C) Tissue distribution of *sid-1* like 1 gene in *S. exigua* of 5^th^ instar larvae. The housekeeping gene *β-actin* was used as internal control.

Using degenerate primers designed on the basis of conserved domain of insect deduced SID-1 amino acid sequences, a partial sequence (231 bp) was obtained from *S. exigua* by RT-PCR, and a longer 939 bp sequence was isolated using 3′-RACE with the specific primers on the 231 bp PCR fragment. Multiple alignment and phylogenetic relationship analysis of deduced amino acids for the SID-1 conserved domain of insects and nematode *C. elegans* SID-1 indicated that the 939 bp fragment (GenBank accession no. **FJ619650**) belongs to a *sid-1* like gene (Figure 1A and S1). The phylogenetic tree showed that *S. exigua* SID-1 like 1 have the closest relationship with lepidopteran *Bombyx mori* SID-1 like 2 deduced proteins (Figure 1A).

To characterize the developmental expression pattern of *sid-1* like gene in *S. exigua*, *sid-1* mRNA levels were detected using RT-PCR method in all developmental stages, including larvae from 1^st^ to 5^th^ instars, prepupae, pupae and adults. The developmental expression pattern of *S. exigua sid-1* showed that this gene was present in all life stages. However, in the prepupae and pupae developmental stages, *sid-1* mRNA exhibited relatively lower expression compared to those in the larvae and adult life stages (Figure 1B). In order to further verify tissue distribution of *sid-1* gene in *S. exigua*, the transcript levels of *sid-1* in cuticle, trachea, midgut and fat body of 1 day 5^th^ instar larvae were detected, and the results indicated that the transcript levels of *sid-1* were similar in the four tissues (Figure 1C).

### Ingestion of bacterially expressed dsRNA reduces *SeCHSA* mRNA level

**Figure 2:**
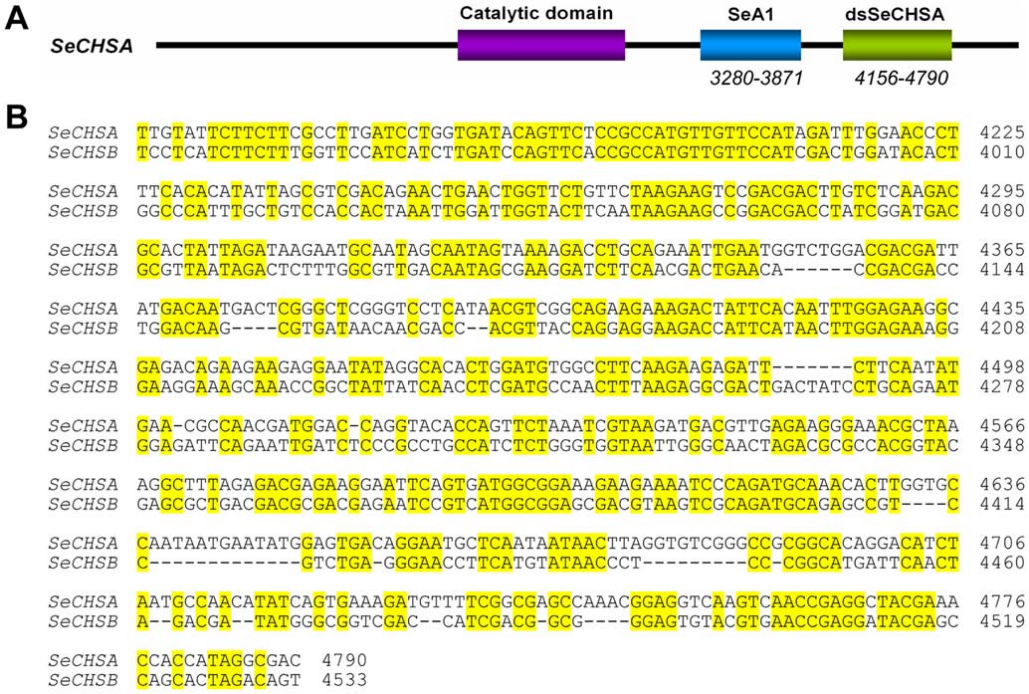
Schematic diagram and nucleotide sequence of *SeCHSA* for dsRNA expression. (A) Schematic diagram of *SeCHSA* mRNA. The dsSeCHSA region (green box) is for dsRNA expression, the SeA1 region (blue box) is for RT-PCR detection, and the red box represents the putative catalytic domain of *SeCHSA*. (B) Alignment of the nucleotide sequences of *SeCHSA* and *SeCHSB* in the region for dsRNA generation.

**Figure 3:**
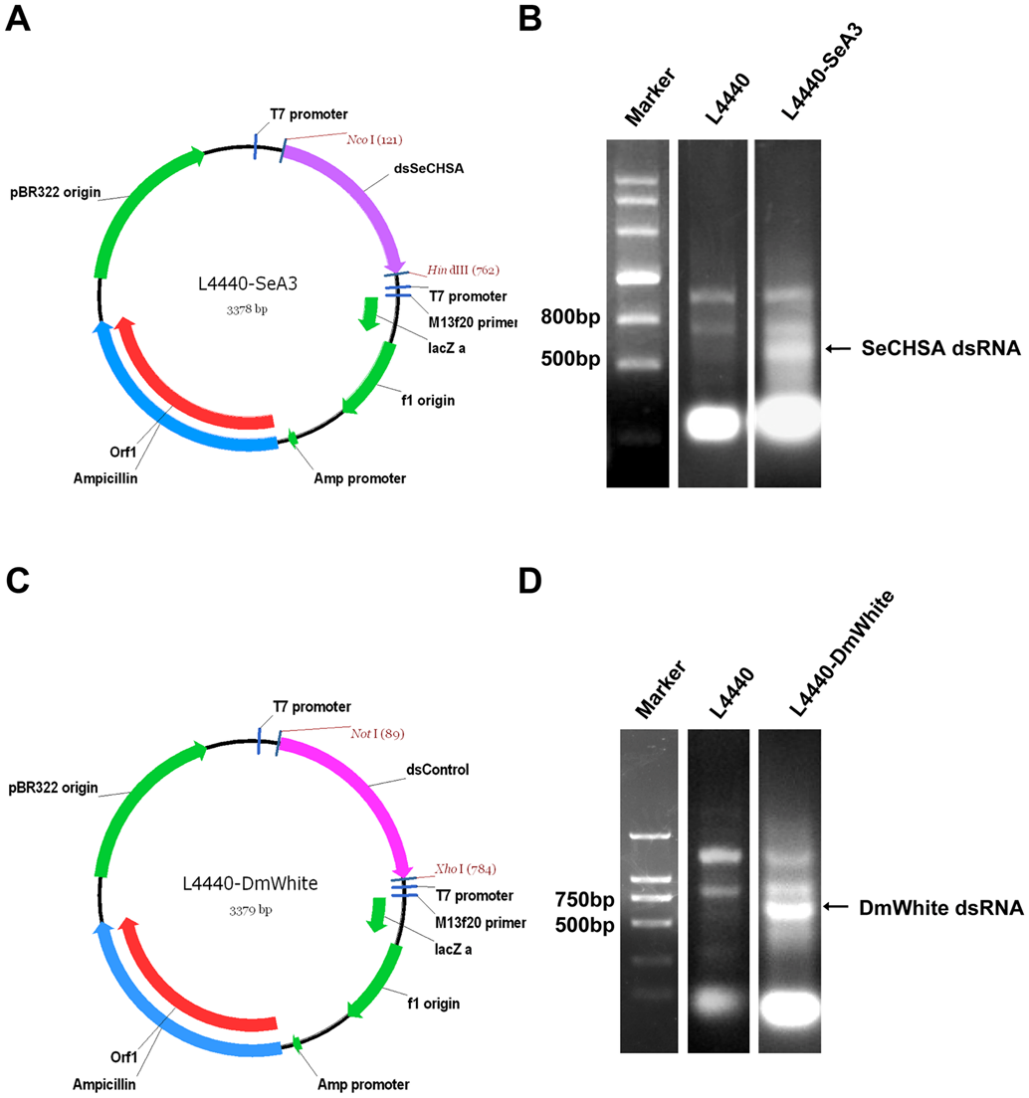
Schematic diagram of the recombinant plasmids for dsRNA expression and production of dsRNAs. (A) Schematic diagram of L4440-SeA3 for *SeCHSA* dsRNA expression. (B) Total RNA was extracted from bacteria HT115(DE3) containing the plasmid L4440-SeA3 and the blank plasmid L4440 after IPTG induction. The position of dsRNA produced is indicated with an arrowhead. (C) Schematic diagram of L4440-DmWhite for *DmWhite* control dsRNA expression. (D) Total RNA was extracted from bacteria. The position of dsRNA produced is indicated with an arrowhead.

Since the important gene *sid-1* for systemic RNAi was proven to be present in *S. exigua*, we constructed a vector expressing a partial sequence of chitin synthase *SeCHSA* (dsSeCHSA) to see whether ingestion of dsRNA to the *SeCHSA* gene, which is not expressed in the midgut, can also reduce target mRNA expression. To verify that the result was specific to RNAi of the *SeCHSA* gene, dsSeCHSA (635 bp, Figure 2A) was aligned with *SeCHSB*; 19 bp consecutive identical sequences were not found (Figure 2B). dsSeCHSA was inserted into the plasmid L4440 within the *Nco*I and *Hin*dIII sites (Figure 3A), and *SeCHSA* dsRNA was produced by bacteria strain HT115(DE3) containing recombinant plasmid L4440-SeA3 (Figure 3B). At the same time, we used a non-related gene, *D. melanogaster white* gene, as a control for dsRNA expression (Figure 3C and D).

**Figure 4:**
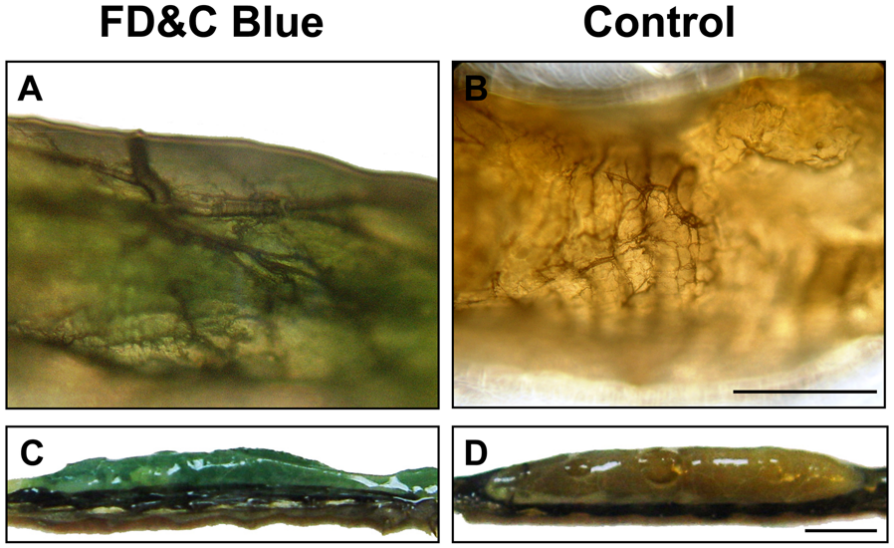
Ingestion and accumulation of bacteria in the midgut of *Spodoptera exigua* larvae. (A) The midgut (slightly blue) from 4^th^ instar larvae fed on the diet containing FD&C Blue colored bacteria for 1 day. (B) The midgut from the larvae fed on normal artificial diet for 1 day. (C) A section of the larval midgut from the larvae fed on the diet containing FD&C Blue colored bacteria for 3 days. (D) A section of the larval midgut from larvae fed on normal artificial diet for 3 days. Scale bars: 1 mm in A and B; 2.5 mm in C and D.

To confirm that bacterially expressed dsRNA was introduced into the larval gut through ingestion, an experiment was performed with *S. exigua* larvae fed artificial diet coated with bacteria that were colored with FD&C Blue. The colored bacteria were easily detectable inside the larvae (Figure 4). After one day of feeding on the diet containing colored bacteria, the blue diet was observed in the midgut of larvae (Figure 4A); blue color accumulated in the midgut two days later (Figure 4C). These results demonstrate that bacteria were ingested by larvae and accumulated in the midgut.

**Figure 5:**
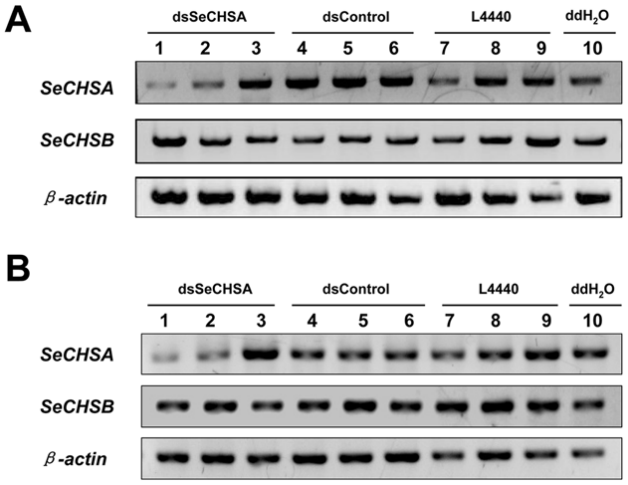
Effects of ingesting bacterially expressed dsRNA on transcription of *SeCHSA* and *SeCHSB.* On day 7 (A) and day 9 (B) post-feeding, total RNA was extracted from individual larvae feeding on the diet containing bacterially expressed *SeCHSA* dsRNA (dsSeCHSA) and *SeCHSA* and *SeCHSB* transcripts were detected using RT-PCR. RNA from larvae fed on the diet containing the control bacteria with *DmWhite* (dsControl), L4440 or with ddH_2_O served as controls. Lanes 1, 4 and 7 represent the larvae fed on high bacteria concentration (250×), lanes 2, 5 and 8 represent results from larvae fed with medium bacteria concentration (50×), and lanes 3, 6, 9 represent larvae fed with low bacteria concentration (10×). The housekeeping gene *β-actin* was used as a reference.

Previous reports showed that ingestion of only high dose dsRNA can induce an RNAi effect. Therefore, in order to select a suitable concentration for RNAi response, we designed a gradient concentration of dsRNA. After expressing dsRNA, bacteria cells were collected and diluted with different volumes of ddH_2_O. The dsRNA concentrations were designated as high dose (250×), medium dose (50×), and low dose (10×) based on the dilution factors. Larvae were fed with bacteria for 3, 5, 7 and 9 days, and RT-PCR was performed to detect the transcript of *SeCHSA*. To avoid possible disturbance of the dsRNA delivered into the larval body, primers were designed for detecting the SeA1 fragment, but not the dsSeCHSA fragment used for dsRNA expression (Figure 2A). The RT-PCR results showed that *SeCHSA* mRNA was substantially decreased on days 7 and 9 in the larvae that ingested high dose dsSeCHSA, compared to the negative controls (dsControl, L4440 and ddH_2_O) (Figure 5A and B). However, larvae fed the medium dose dsSeCHSA showed only a slight decrease in *SeCHSA* transcript levels, and no change in *SeCHSA* transcript was found in larvae fed on low dose dsSeCHSA (Figure 5A and B). These results suggested that only a sufficient concentration of dsRNA can induce target gene knockdown. In addition, expression of a midgut specific chitin synthase gene (*SeCHSB*) in *S. exigua* larvae was not affected by feeding with dsSeCHSA at different concentrations (Figure 5A and B), indicating that the observed RNAi was gene specific. We did not detect any variation in *SeCHSA* mRNA levels after ingestion of dsRNA for 3 and 5 days, even with high dose dsSeCHSA (Figure S2). These results suggested that the RNAi response induced by ingestion of dsRNA requires an accumulation of dsRNA in the larvae.

**Figure 6:**
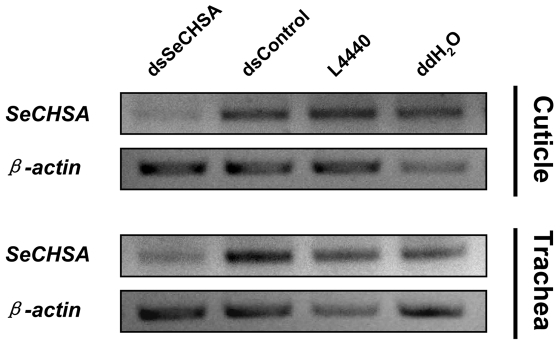
Effects of ingesting bacterially expressed dsRNA on *SeCHSA* mRNA in the cuticle and trachea. Total RNA was extracted from the cuticle and trachea tissues respectively after feeding with bacterially expressed dsRNA (the high bacteria concentration, 250×) for 9 days, ddH_2_O and bacteria containing *DmWhite* dsRNA (dsControl) or blank plasmid L4440 served as controls. *SeCHSA* transcript was detected using RT-PCR, *S. exigua β-actin* gene was used as a reference.

Because *SeCHSA* was found to be expressed in the cuticle and trachea [17], after the observation of specific transcript levels decreasing through ingestion of high dose dsSeCHSA in *S. exigua*, a further examination was performed to analyze the *SeCHSA* mRNA levels in cuticle and trachea after 9 days feeding with high dose dsSeCHSA. The results showed that the transcript levels of *SeCHSA* decreased in cuticle and trachea compared to the controls (Figure 6). The target gene knockdown in cuticle and trachea suggested that systemic RNAi can be induced through ingestion of dsRNA for a non-midgut gene in *S. exigua*.

### RNAi by feeding can control larval development

**Figure 7:**
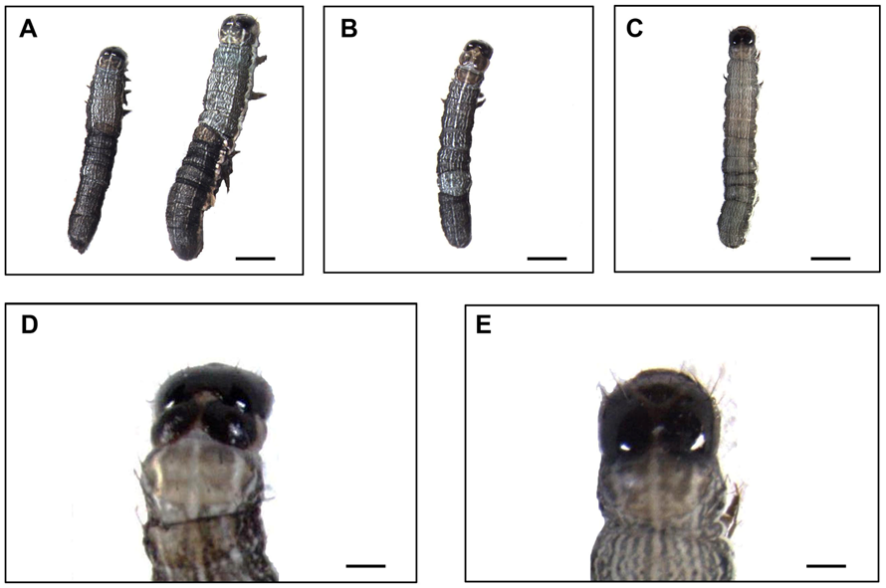
Phenotypes of *Spodoptera exigua* after ingestion of bacterially expressed dsRNA (high bacteria concentration, 250×). (A) The “half-ecdysis” phenotypes of 4^th^ instar (left) and 5^th^ instar (right) larvae after feeding on bacterially expressed *SeCHSA* dsRNA. (B) A larva with “half-ecdysis” phenotype has a “double heads”. (C) The normal phenotype of a 4^th^ instar larva fed with control bacteria dsControl, L4440 or ddH_2_O. (D) The enlarging “double-heads” of (B). (E) The enlarging normal head of (C). Scale bars represent 2 mm in A, B and C, and 0.4 mm in D and E.

After observing specific gene silencing following ingestion of bacterially expressed dsSeCHSA, we were interested to determine whether inhibition of the *SeCHSA* mRNA expression leads to a lethal phenotype in *S. exigua* larvae. Larvae were fed a diet containing bacterially expressed dsRNA to *SeCHSA* at day 1 of the 1^st^ instar, and continuously fed until the 3^rd^ day of 5^th^ instar, using three different dsRNA concentrations. Prior to the 3^rd^ instar, no interference phenotype was observed. After continuous feeding with the dsSeCHSA-containing diet for eight days, we observed that about 25.7% of individuals fed on high dose (250×) dsSeCHSA exhibited an obvious “half-ecdysis” phenotype at early 4^th^ instars or between the 4^th^ and 5^th^ instars, especially during the larval molting process (Figure 7A and B). Approximately 10% of those “half-ecdysis” larvae developed a “double heads” phenotype (Figure 7B and D). These “half ecdysis” phenotype larvae usually could remove only a part of their old cuticle, always failed to complete ecdysis, and died within two days. On the contrary, nearly all the larvae fed with medium and low doses of dsSeCHSA, as well as control larvae, molted normally into the next stage. This RNAi-mediated malformed phenotype correlates with the decreased mRNA levels of *SeCHSA* in the larvae fed on diets containing bacteria-expressed dsSeCHSA (Figure 5). However, no abnormal phenotype was observed from larvae to prepupae, prepupae to pupae, or pupae to adult, even after continuous ingestion of bacterially expressed dsSeCHSA (data not shown). In addition, nearly all remaining living 5^th^ instar larvae, prepupae and pupae developed normally, showing no significant differences from controls. During the stage of prepupae to pupae, a small number of larvae fed on dsRNA-containing diet exhibited variable malformed pupae (Figure S3); this was also observed in control larvae. Thus, this abnormal phenotype appears not to be a result of RNAi. The phenotypes observed following ingestion of the *SeCHSA* dsRNA are similar to those caused by benzoylphenyl ureas, which are insect growth regulators [23]. This suggests that the mechanisms of action of the dsRNA and of the ureas may be similar.

**Figure 8:**
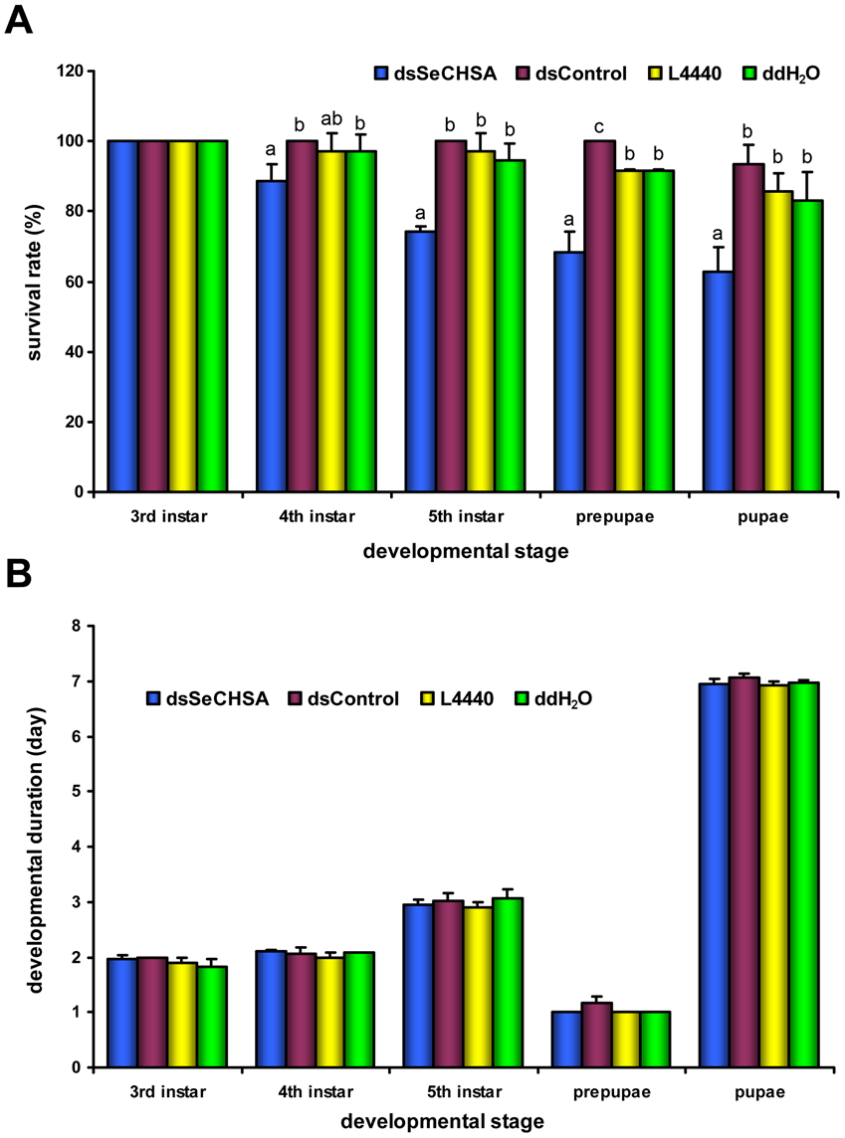
Effects of ingested dsSeCHSA on *Spodoptera exigua* survival and developmental duration. (A) Survival rates of insects at different developmental stages after ingestion of dsSeCHSA (the high bacteria concentration, 250×). Different letters in the same developmental stage indicate significant difference of the survival rates (p<0.05, Duncan's test). (B) Developmental duration of the insects after ingestion of dsSeCHSA. No significant difference was found by ANOVA (p>0.05). All error bars represent standard deviation (n = 3).

In the 4^th^ and 5^th^ instars larvae, prepupae, and pupae, the average survival rates of the insects fed on the high dose dsSeCHSA-containing diet were 88.64%, 74.24%, 68.43%, and 62.63%, respectively (Figure 8A). The survival rates in the 5^th^ instar larvae, prepupae and pupae stages were significantly lower (p<0.05, Duncan's test) than those of all controls, and significant lethality differences were also found between dsSeCHSA treatment and dsControl or ddH_2_O control in the 4^th^ instar larvae. In addition, more than 80% of the dead larvae in the high dose dsSeCHSA feeding group exhibited the “half-ecdysis” phenotype. However, no significant differences were found in the developmental duration of the insects at different developmental stages after ingestion of the high dose dsSeCHSA (p>0.05, ANOVA) (Figure 8B). In the larvae fed on medium (50×) and low doses (10×) of dsSeCHSA or control larvae, no obvious differences were found in the survival rate or development duration (data not shown).

### Knockdown of *SeCHSA* expression disrupts larval cuticle structure

Our previous study showed that *SeCHSA* is expressed in the cuticle, trachea, and midgut (possibly due to contamination from trachea) by Northern blot analysis [17]. Abnormal larvae injected with siRNA or dsRNA to the *SeCHSA* gene contain disordered chitin layers in the cuticle [20]. Thus, we set out to determine whether ingestion of dsSeCHSA affects the structure of larval cuticle, trachea or midgut peritrophic matrix in abnormal larvae.

**Figure 9:**
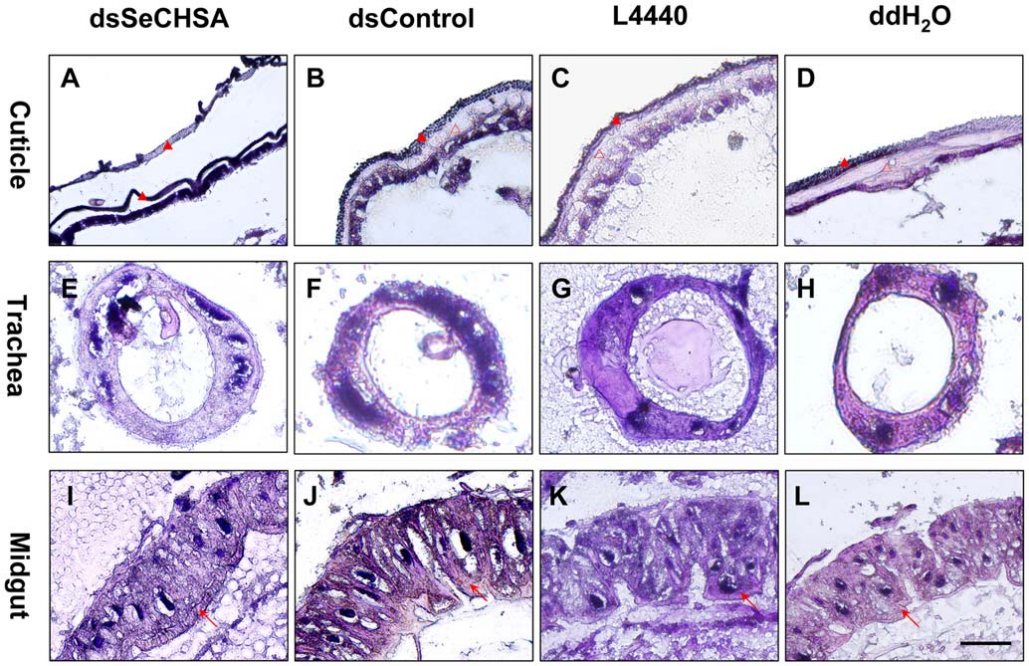
Effects of ingested dsSeCHSA on different tissues of *Spodoptera exigua* larvae. Cuticle, trachea and midgut peritrophic matrix were dissected from the abnormal larvae 9 days after feeding on the diet containing bacterially-expressed *SeCHSA* dsRNA (dsSeCHSA, the high bacteria concentration, 250×), or from the larvae at the same stage fed on bacterially-expressed *DmWhite* dsRNA (dsControl), control L4440 bacteria or ddH_2_O. All tissues were stained with Hemotoxylin and eosin (H & E). The filled and open red triangles in A, B, C and D represent exocuticle and endocuticle, respectively; the red arrows in I, J, K and L represent peritrophic matrix of the midgut. Scale bar represents 50 µm.

Hematoxylin and eosin (H & E) staining was used to investigate the histological structure of abnormal 4th instar day two larvae that had been fed the dsSeCHSA-containing diet. In abnormal larvae (high dose dsSeCHSA feeding group), the integral structure of the cuticle was disordered, particularly in the endocuticle, with a double exocuticle present (Figure 9A); the cuticles of the controls were well-structured (Figure 9B, C and D). Clear peritrophic matrix of midgut was observed in both abnormal and control larvae (Figure 9I, J, K and L), and no structural differences were found in the trachea (Figure 9E, F, G and H). These results prove that *SeCHSA* affects chitin synthesis in the cuticle. In addition, this result further supports our previous suggestion that apparent *SeCHSA* expression in the midgut may be due to contamination of the tracheae surrounding the midgut [17]. However, even though slight *SeCHSA* mRNA knockdown was found in medium dose dsSeCHSA feeding larvae (Figure 5), we didn't find any significant differences neither in the cuticle nor in the peritrophic matrix of midgut or trachea compared with that of the controls (Figure S4); the similar results were also observed in the larvae ingestion of low dose dsSeCHSA (Figure S5). These results may suggest that only sufficient *SeCHSA* transcriptional level decreasing can do effect on the normal histological structure of *S. exigua*.

## Discussion

### Ingestion of bacterially expressed *SeCHSA* dsRNA induces lethal phenotypes in *S. exigua* larvae

In this study, we demonstrated that ingestion of bacterially expressed dsRNA can specifically inhibit transcription of a targeted gene in the lepidopteran insect *S. exigua*. Knockdown of the target gene *SeCHSA* not only suppresses its transcription levels, but also affects larval growth and development, leading to lethal phenotypes. A previous study in the lepidopteran *Spodoptera litura* using ingestion of dsRNA did not induce an RNAi response [24]. Moreover, oral delivery of dsRNA to larvae of the lepidopteran *Epiphya postvittana* did not cause mortality, in spite of reduction in the transcript levels of the target gene [10]. Similarly, in the hemipteran *Rhodnius prolixus*, ingestion of dsRNA (13 µg per larva) for a salivary nitrophorin 2 gene reduced the target gene expression without any significant changes in salivary gland color [25]. More recently, food intake by larvae of the orthopteran *Gryllus bimaculatus* that were fed on dsRNA (6 µg per larva) of a nervous system neuropeptide gene *SK* was stimulated [11]. Also, ingestion of artificial diet containing dsRNA of a midgut gene *V-ATPase A* in larvae of the coleopteran insect western corn rootworm (WCR) *Diabrotica virgifera virgifera* resulted in larval stunting and mortality; remarkably, transgenic corn plants engineered to express WCR dsRNAs exhibited a significant decreasing in WCR feeding damage in the growing chamber assay [7]. At the same time, another research group reported that, when a lepidopteran cotton bollworm *Helicoverpa armigera* larvae were fed with plant expressed dsRNA specific for a cytochrome P450 gene (*CYP6AE14*), not only the transcript levels of the gene reduced in the midgut but also the larval growth was retarded [8]. However, it is still unclear whether some insecticide phenotypic silencing can be observed for genes not expressed in the midgut through ingestible dsRNA. We showed that ingestion of dsRNA for a gene (*SeCHSA*) that is not expressed in the midgut also caused a lethal larval phenotype.

Our results suggest that dsRNA introduced into the midgut of larvae may be transported to other insect cells or tissues; this process may occur through the intercellular dsRNA transport protein SID-1 [2], [10]. SID-1 was first identified in *C. elegans*, has been proven to be required for spreading RNA interfering information between tissues, and usually leads to systemic RNAi [21]. To determine whether SID-1 exists in *S. exigua*, we cloned a partial sequence of *sid-1* like gene from the beet armyworm, which shares a high identity (70%) with *B. mori sid-1* like 2 gene (GenBank accession no. **AB327185**) and *C. elegans sid-1* gene (46.8%) (GenBank accession no. **NM_071971**); and this was consistent with the phylogenetic relationship analysis of SID-1 in insects and *C. elegans* (Figure 1A). The expression pattern analysis of *sid-1* gene suggested that it expressed in all the developmental stages (Figure 1B), and also it exhibited a similar expression levels in the four tissues of cuticle, trachea, midgut and fat body in *S. exigua* (Figure 1C). Existence of this systemic RNAi vital gene transcript in all larval developmental stages and four tissues of *S. exigua* may account for the systemic RNAi effect in this insect after ingestion of dsRNA. To our knowledge, this is the first report that ingestion of bacterially expressed non-midgut gene dsRNA can cause lethal larval phenotypes in lepidopteran insect.

We also showed that larval-larval ecdysis was disrupted in *S. exigua* larvae fed a diet containing bacterially expressed *SeCHSA* dsRNA. Abnormal larvae that ingested dsSeCHSA often failed to remove their old cuticle completely and about 10% of them could not remove their head capsule (Figure 7). These phenotypes are similar to those observed in insects treated with acylureas [23]. As insect growth regulators, acylureas selectively disrupt chitin synthesis in insects but not in fungi, and they have played a significant role in IPM (Integrated Pest Management) for more than 30 years [16], [18]. Bacteria expressing dsRNA may be used in insect pest control in the near future, since crude extracts of bacterially expressed dsRNA effectively protect plants from virus infections [26].

Closer examination of the cuticle in the abnormal *S. exigua* larvae using H & E staining revealed that the exo- and endocuticle of the abnormal larvae were disordered (Figure 9A). This result suggests that *SeCHSA* is essential for larval-larval growth and development, an observation which is partly in agreement with phenotypes observed after injection of dsRNA to *TcCHS1* in the red flour beetle *Tribolium castaneum* [27]. However, we did not find any significant changes in the phenotype during larval-pupal and pupal-adult stages after ingestion of bacteria-expressed dsSeCHSA in *S. exigua* (data not shown). This observation also differed from results in *T. castaneum*, in which *T. castaneum* larvae injected with dsRNA in the penultimate larval instar failed to pupate, and larvae injected in the last instars did not complete pupal development, although larval-pupal molting was initiated [27]. One possible reason that distinct changes in the phenotypes during larval-pupal development were not observed in our study is that the dsRNA ingested into the last instar larval gut may not have reached levels high enough to cause changes in phenotype. Another possible explanation is that RNAi in lepidopteran insects may not be as effective as in the coleopteran beetle *T. castaneum*, in which dsRNA-mediated responses can be easily observed even with an injection of as little as 5 pg dsRNA [28]. The amount of dsRNA injected into lepidopteran insects in various studies has ranged from 0.1 µg to 6 µg per larva [10], [29], [30] and up to 20 µg per pupa in the silkworm *B. mori* [31].

Although the transcription of *SeCHSA* in the trachea was down-regulated after ingestion of dsSeCHSA (Figure 6), no structural difference was found in the trachea (Figure 9E, F, G and H). One possible reason is that the trachea used here was the cross section of main trachea tube originated from the spiracle, for the hardly observing any tracheole in the larvae using histological method, little knockdown of the *SeCHSA* transcript couldn't result in abnormal trachea in structure. In addition, we have found that amount of tracheole in the abnormal larvae treated with dsSeCHSA was less than that in normal larvae to a certain degree. It suggests that tracheole may be affected by ingestion of dsRNA, which is consistent with our previous report the tiny trachea defect was observed using dsRNA injection method for the same gene [20].

### Delivery of dsRNA by feeding is a convenient and reliable RNAi method, especially for gene screening

Several methods for the delivery of dsRNA or siRNA have been used in recent years to knock down specific gene expression. Direct microinjection remains the most popular method for its simple protocol and effectiveness in knocking down expression of target genes, including the homothorax gene and the evenskipped gene in the cricket *G. bimaculatus* [32], [33], the chitin synthesis gene in the midgut of the mosquito *Aedes aegypti* [34], three *BgEcR* genes in the hemimetabolous species *Blattella germanica* [35], [36], and a bursicon gene in the silkworm *B. mori* [31]. However, it is very difficult to achieve RNAi via injection for small insects like aphids or early instar larvae or nymphs, where injection may result in high mortality. In addition, it usually requires special techniques and equipment.

Delivery of dsRNA by feeding has several advantages over microinjection because feeding is less labor-intensive and less expensive and is extremely convenient when performing RNAi on a large number of genes and individuals [9]. Ingestion of dsRNA has been proven to confer lethal phenotypes by silencing midgut-specific genes. Recent studies have shown that transgenic plants expressing insect dsRNA can be exploited to control insect pests [2], [7], [8]. However, identification of ideal genes for inhibition in transgenic plants is needed. Our results suggest that delivery of dsRNA by feeding is a convenient and reliable method for screening a large number of essential insect genes, as reported in beetles and termites [7], [12].

### *SeCHSA* is a good target gene for RNAi application in pest control

Insect-resistant transgenic plants have been in commercial use for more than 10 years. Theoretically, it is better to use genes that originate from insects as transgenes, but this has not yet been realized. Damage caused by insect pests is reduced in plants that express an insect dsRNA [7]. This presents an opportunity to combine the efforts of entomological research with the biotechnology industry [2]. In order for this approach to be successful, suitable insect genes for transgenic plants must be discovered. In addition, it is necessary for transgenic plants expressing insect dsRNA to be both safe and effective. Gene screening is an important step in this process.

Chitin is the most widespread natural amino polysaccharide. Since chitin synthesis is crucial for growth and development in insects, and chitin is not found in plants and vertebrates, chitin synthesis has been deemed an ideal target for novel insecticides since the 1970s [16], [18]. Our results show that feeding *S. exigua* with dsSeCHSA has the potential to act as an insecticide, and suggest that chitin synthase *SeCHSA* is an ideal target gene for pest control. Already, transgenic plants expressing *SeCHSA* dsRNA have been produced in our lab, and safety testing of the transgenic plants in vertebrates and higher animals is currently underway.

## Materials and Methods

### Insect culture

Larvae of the beet armyworm, *S. exigua*, were obtained from Nanjing Agricultural University and were reared in the laboratory at 25°C±2°C and 75%±5% relative humidity on a 14 hr-light/10 hr-dark photoperiod using an artificial diet [37].

### Cloning of *sid-1* like gene fragment and its expression pattern analysis

Total RNA was extracted from the 5^th^ instar larvae of *S. exigua* using Trizol reagent (Invitrogen) and cDNA was synthesized using the AMV reverse transcriptase XL (Takara) as the following reaction conditions: 37°C for 10 min, 42°C for 1 h, 99°C for 5 min, and 5°C for 5 min. In order to clone *S. exigua sid-1* gene, degenerate primers based on the conserved amino acids FNHVFSN (forward primer) and NFQFDT (reverse primer) were used to get a fragment of *sid-1* gene. The primers used were as follows: SID1F1 (5′- TTY AAY CAY GTN TTY WSN AA-3′, forward primer), and SID1R1 (5′-NTG RTC RAA YTG RAA RTT- 3′, reverse primer). PCR amplification with primers SID1F1 and SID1R1 was performed as following: 94°C for 5 min, 5 cycles of 94°C for 40 s, 58°C for 40 s and 72°C for 40 s, 25 cycles of 94°C for 40 s, 55°C for 40 s and 72°C for 40 s, and with an extension step at 72°C for 10 min at the end. The amplified fragment was subcloned into the pMD-18T vector (Takara) and sequenced. To get a longer cDNA sequence, we performed 3′-RACE using gene specific primers for *sid-1* and anchor primers supplied in a SMART RACE cDNA Amplification Kit (Clontech). For 3′-RACE, the gene specific primers that were designed based on the partial amplified fragment mentioned above, a forward primer and a nested primer were: SID1-GSP2 (5′-CTG GGA TAT GTG CTA CTA GGC CTG C-3′), SID1-NGSP2 (5′-GCT GGG TAT ACC ACA GCA TTT TGG CC-3′), respectively.

The methods for total RNA extraction and cDNA synthesis were the same as described above. For analysis of the *sid-1* mRNA in different developmental stages, total RNA was extracted from 1^st^ to 5^th^ instars larvae, prepupae, pupae and adult. For analysis of *sid-1* mRNA in different tissues, RNA was extracted from the cuticle, trachea, midgut and fat body of 1 day 5^th^ instar larvae. The primers used for sid-1 expression pattern analysis were forward primer SID1-GSP2 as described above and reverse primer SID1-GSP1.1 (5′- CAG GCA GGT GGC GAG GTG GAG GAC C-3′) as described, this pair of primers spanned a nucleotide fragment of 402 bp. Amplification reactions comprised 30 cycles of 94°C for 401 s, 55°C for 40 s, and 72°C for 40 s, with a final extension step of 72°C for 10 min.

The deduced amino acids of *S. exigua* SID-1, other insect SID-1 and nematode *C. elegans* SID-1, the deduced amino acids of conserved domain were aligned by ClustalW. Phylogenetic analysis were conducted by the neighbour-joining method using MEGA version 4.0 [38]. Bootstrap values were assessed with 1000 replicates. The deduced amino acid sequences of insect SID-1 and *C. elegans* SID-1 were obtained from GenBank. GenBank accession numbers of the deduced sequences used as the following: *S. exigua* SID-1 like 1, **ACM47363**; *B. mori* SID-1 like 1, **BAF95805**; *B. mori* SID-1 like 2, **BAF95807**; *B. mori* SID-1 like 3, **BAF95806**; *T. castaneum* SID-1 related A, **NP_001099012**; *T. castaneum* SID-1 related B, **NP_001103253**; *T. castaneum* SID-1 related C, **NP_001099128**; *A. mellifera* SID-1 like, **XP_395167**; and *C. elegans* SID-1, **AAL78657**.

### Vector construction and dsRNA preparation

In order to construct a plasmid that expresses dsRNA corresponding to *S. exigua* chitin synthase A (*SeCHSA*, GenBank accession no. **DQ062153**), a 635 bp fragment (dsSeCHSA) was amplified by RT-PCR using total RNA as a template. The forward primer was SeA3F (5′-TCG CCATGG TTG TAT TCT TCT TCG CCT TG-3′), which spans nucleotides 4156–4175, and the reverse primer was SeA3R (5′-GAC AAGCTT GTC GCC TAT GGT GGT TTC GT-3′), which spans nucleotides 4790–4771. The underlined portions of sequence are *Nco*I and *Hin*dIII restriction sites, respectively. Amplification reactions comprised 30 cycles of 94°C for 40 s, 58°C for 40 s, and 72°C for 40 s, with a final extension step of 72°C for 8 min. PCR products were confirmed by separation on 1% agarose gels and visualized by ethidium bromide staining. The PCR product was then cloned into the plasmid L4440 [4] between the *Nco*I and *Hin*dIII sites. The L4440 plasmid, which was provided by Andrew Fire (Stanford University, CA, USA) and obtained from Addgene Inc. (Cambridge, MA, USA), has two T7 promoters in inverted orientation flanking the multiple cloning site [13]. The resulting recombinant vector L4440-SeA3 was introduced into competent HT115(DE3) cells [4], [9] lacking RNase III. The HT115(DE3) bacterium has the following genotype: F-, mcrA, mcrB, IN (rrnD-rrnE)1, rnc14::Tn10 (DE3 lysogen: lavUV5 promoter -T7 polymerase); the RNase III gene is disrupted by a Tn10 transposon carrying a tetracycline-resistance marker [9]. This bacterium can be induced to express T7 polymerase in the presence of isopropyl β-D-thiogalactoside (IPTG). The HT115(DE3) was provided by Lisa Timmons (University of Kansas, KS, USA) & Andrew Fire and obtained from CGC (Caenorhabditis Genetics Center, Minneapolis, MN, USA).

To produce dsRNA, single colonies of HT115(DE3) bacteria containing dsSeCHSA or cloned L4440 were grown for 14 h with shaking in LB with 100 µg/ml ampicillin plus 12.5 µg/ml tetracycline at 37°C. The culture was diluted 100-fold in 100 ml of 2×YT medium and allowed to grow to OD_595_ = 0.4. Synthesis of T7 polymerase was induced by addition of IPTG to 0.4 mM and the bacteria were incubated with shaking for an additional 4 h at 37°C. The expressed dsRNA was extracted as described previously [4], [26]. The length of the dsRNA was confirmed by electrophoresis on 1% agarose gel. To prepare bacterial cells that express dsRNA, the procedures described above were used. Bacterial cells were collected from 100 ml IPTG-induced culture by centrifugation at 10,000 g for 2 min, resuspended in 0.4 ml (250×), 2 ml (50×) or 10 ml (10×) sterile water, and then used for *S. exigua* feeding bioassays.

An unrelated gene of the *D. melanogaster, white* gene (*DmWhite*) (GenBank accession no. **X51749**), was selected for use as a control dsRNA. The recombinant plasmid for *DmWhite* dsRNA expression protocol was the same as that for dsSeCHSA. The PCR primers used to amplify the fragment (688 bp) of *DmWhite* were as follows: forward primer DmWhiteF (5′-GG CTCGAG ATG GGC TAC CGG CGC CCA GGA AAC ATT-3′, spanning nucleotides 322–342; underlined letters indicate a *Xho*I site) and reverse primer DmWhiteR (5′-GG GCGGCCGC TAG GAA AAG AAG TCG ACG GCT TCG C-3′, spanning nucleotides 1009–985; underlined letters indicate a *Nto*I site).

### Feeding bioassays

A randomized block design was used for this feeding experiment. The artificial diet pellets were cut into two sizes, the larger one about 20 mm×10 mm×2 mm and about 0.9 g in weight for *S. exigua* neonates, 1^st^ and 2^nd^ instars larvae, and a smaller one about 10 mm×10 mm×2 mm and about 0.45 g in weight for 3^rd^–5^th^ instars larvae. Each piece of diet was overlaid with a 50 µl suspension of one of the following: bacterial culture containing bacteria expressing dsRNA for *SeCHSA*, bacterial culture containing bacteria expressing control dsRNA for *DmWhite*, plasmid L4440, or 50 µl ddH_2_O. For experiments employing different concentrations (250×, 50×, 10×) the feeding protocols were the same as described above. Prior to the 3^rd^ instar, *S. exigua* neonates and larvae were reared on four pieces of the diet in a controlled chamber as groups in the same conditions as described above. Ten to twelve day one 3^rd^ instar larvae were selected by weight (about 0.0025 g with 5 mm body length) from each experimental group or from the control group was subsequently reared individually on one piece of the diet for each condition. Each group was replicated three times. All diets were replaced daily. Insect molting, survival, and abnormal phenotype were observed and recorded daily for each individual insect until death. Five normally-developed larvae randomly selected from the experimental or the control groups were also weighed daily using an electronic scale (0.0001 g) (Mettler Toledo, AB104-S, Switzerland) from 3^rd^ instar day one to 5^th^ instar day two. Data on survival rate (arcsine square root transformed), weight and developmental duration were analyzed by one-way analysis of variance (ANOVA). When treatment effects were detected, Duncan's test was used to determine whether significant differences exist among the survival rates.

To confirm that bacteria were ingested by larvae, FD&C Blue (Lab of Dr. Ehrenstorfer, Augsburg, Germany) was used to color the bacteria that were overlaid onto the surface of the insect artificial diet. Fourth instar day two larvae were allowed to feed on the colored diet. One day after feeding on the diet containing FD&C Blue, the larvae were dissected and their midguts were examined with a Carl Zeiss Axiostar plus microscope (Göttingen, Germany). To determine whether the colored bacteria accumulated in the midgut, the midguts were dissected for observation. All samples were photographed using a Sony DSC-F717 digital camera.

### RT-PCR analysis to confirm gene silencing

To monitor transcriptional levels of *SeCHSA* in larvae after feeding with bacteria which express dsRNA, total RNA was extracted from the experimental and control larvae using Trizol reagent (Invitrogen) according to the manufacturer's instructions. Each RNA sample (0.5–1 µg) was used for cDNA synthesis in a 40 µl reaction mixture using the following reaction conditions: 37°C for 10 min, 42°C for 1 h, 99°C for 5 min, and 5°C for 5 min. For PCR reactions, 2 µl of *S. exigua* cDNA was used as a template in 50 µl reactions using *SeCHSA* specific primers. The forward primer was SeA1F (5′-TAC CCT CTA TGT ACT TGC TT-3′, spanning nucleotides 3280–3299) and the reverse primer was SeA1R (5′-AAA GAT AAT TCG GCG GGA CT-3′, spanning nucleotides 3871–3852). The PCR amplification conditions were the same as described above. In order to determine whether RNAi of the *SeCHSA* gene affects the expression of *S. exigua* chitin synthase gene B, a pair of primers for *SeCHSB* (GenBank accession no. **EU622827**) were designed as follows: forward primer SeCHSB1F (5′-TTA CGT CAC CAT TCC CAG C-3′, spanning nucleotides 3114–3132), and reverse primer SeCHSB1R (5′-AAG TTA GTC TCT GCC GTC G-3′, spanning nucleotides 3656–3638). The PCR amplification with primers SeCHSB1F and SeCHSB1R were performed as follows: 94°C for 5 min, 30 cycles of 94°C for 40 s, 55°C for 40 s and 72°C for 40 s; and 72°C for 10 min at the end. As a control, the *S. exigua* β-actin (GenBank accession no. **AY507963**) transcript was amplified by PCR using the same cDNA template and actin-specific primers, Se-actinF (5′-GGT TGG TAT GGG TCA GAA GGA-3′, spanning nucleotides 200–220) and Se-actinR (5′-GCG GTG GTG GTG AAA GAG TA-3′, spanning nucleotides 676–657). The PCR reaction conditions were the same as those described previously [17]. The RT-PCR products were analyzed by 1% agarose gel electrophoresis and the gel was stained with ethidium bromide. To further analyze the *SeCHSA* transcriptional levels in the cuticle and trachea after ingestion of high dose dsSeCHSA expressing bacteria for nine days, total RNA was extracted from corresponding tissues as the same methods described above. The same primers and PCR program were used to analysis *SeCHSA* expression levels using RT-PCR as described above.

### Microscopy and histological examination

To determine the impact of gene silencing on the development of *S. exigua* cuticle, trachea and midgut peritrophic matrix, five abnormal larvae (4^th^ instar) were selected nine days after feeding on the dsRNA-containing diet, and same number of larvae were randomly selected from the control groups. These larvae were fixed for 4 h with Carnoy (6 ethanol: 1 acetic acid: 3 chloroform), dehydrated through an ethanol series and xylene, and embedded in paraffin. The larvae were sectioned at 7 µm using a Baffalo microtome (American Optical Company, New York, USA) and stained with hematoxylin and eosin (H & E). All samples were examined with an Olympus research inverted system microscope IX71 connected to a color digital camera Olympus DP71. Digital images were captured using Image Pro Plus (Media Cybernetics, Silver Spring, MD) and processed using Microsoft Office Picture Manager (Microsoft Corporation, USA) and Adobe Photoshop CS to enhance contrast and brightness.

## Supporting Information

Figure S1 Multiple alignment of Spodoptera exigua SID-1 and other insects or nematode Caenorhabditis elegans SID-1 translated amino acid sequences. S. exigua SID-1 like 1, GenBank accession no. ACM47363; B. mori SID-1 like 1, BAF95805; B. mori SID-1 like 2, BAF95807; B. mori SID-1 like 3, BAF95806; T. castaneum SID-1 related A, NP_001099012; T. castaneum SID-1 related B, NP_001103253; T. castaneum SID-1 related C, NP_001099128; A. mellifera SID-1 like, XP_395167; and C. elegans SID-1, AAL78657.
(1.21 MB TIF)

Figure S2 Effects of ingestion of bacteria-expressed dsRNA on transcription of SeCHSA and SeCHSB on day 3 (A) and day 5 (B) post-feeding. Total RNA was extracted from individual larvae feeding on the diet containing bacteria-expressed dsRNA of dsSeCHSA and SeCHSA and SeCHSB transcripts were detected using RT-PCR. RNA from larvae fed on the diet containing the control bacteria with DmWhite (dsControl), L4440 or with ddH2O served as controls. The lane 1, 4 and 7 represent the high bacteria concentration (250×) feeding larvae,the lane 2, 5 and 8 represent the middle bacteria concentration (50×) feeding larvae, and the lane 3, 6, 9 represent the low bacteria concentration (10×) feeding larvae. The house keeping gene β-actin was used as a reference.
(0.25 MB TIF)

Figure S3 The variable malformed pupae after ingestion of high dose dsRNA in Spodoptera exigua. The frenquency of these phenotypes were nearly the same between dsRNA feeding and controls. A, B and C show the variable phenotypes of pupae that appeared in each group. D shows the normal pupae. The scale bar represents 2 mm.
(0.56 MB TIF)

Figure S4 The Effects of ingestion of bacterially-expressed dsRNA (the medium bacteria concentration, 50×) on the cuticle, trachea and midgut peritrophic matrix of Spodoptera exigua larvae. Cuticle, trachea and midgut peritrophic matrix were dissected from the abnormal larvae 9 days after feeding on the diet containing bacterially-expressed SeCHSA dsRNA,or from the larvae at the same stage fed on bacterially-expressed DmWhite dsRNA (Control), control L4440 bacteria or ddH2O. All tissues were stained with Hemotoxylin and eosin (H & E). The filled and open red triangles in A, B, C and D represent exocuticle and endocuticle, respectively; the red arrows in I, J, K and L represent peritrophic matrix of the midgut. Scale bar represents 50 µm.
(1.20 MB TIF)

Figure S5 The Effects of ingestion of bacterially-expressed dsRNA (the low bacteria concentration, 10×) on the cuticle, trachea and midgut peritrophic matrix of Spodoptera exigua larvae. Cuticle, trachea and midgut peritrophic matrix were dissected from the abnormal larvae 9 days after feeding on the diet containing bacterially-expressed SeCHSA dsRNA,or from the larvae at the same stage fed on bacterially-expressed DmWhite dsRNA (Control), control L4440 bacteria or ddH2O. All tissues were stained with Hemotoxylin and eosin (H & E). The filled and open red triangles in A, B, C and D represent exocuticle and endocuticle, respectively; the red arrows in I, J, K and L represent peritrophic matrix of the midgut. Scale bar represents 50 µm.
(1.19 MB TIF)

## References

[1] Kooter JM, Matzke MA, Meyer P (1999). Listening to the silent genes: transgene silencing, gene regulation and pathogen control.. Trends Plant Sci.

[2] Gordon KHJ, Waterhouse PM (2007). RNAi for insect-proof plants.. Nat Biotech.

[3] Orii H, Mochii M, Watanabe K (2003). A simple “soaking method” for RNA interference in the planarian *Dugesia japonica*.. Dev Genes Evol.

[4] Timmons L, Court D, Fire A (2001). Ingestion of bacterially expressed dsRNAs can produce specific and potent genetic interference in *Caenorhabditis elegans*.. Gene.

[5] Britt-Louise L, Eugene IS, Jan B, Elisabeth W, Petra vR (2006). dsRNA-mediated resistance to Beet Necrotic Yellow Vein Virus infections in sugar beet (*Beta vulgaris L. ssp. vulgaris*).. Mol Breeding.

[6] Niu QW, Lin SS, Reyes JL, Chen KC, Wu HW (2006). Expression of artificial microRNAs in transgenic *Arabidopsis thaliana* confers virus resistance.. Nat Biotech.

[7] Baum JA, Bogaert T, Clinton W, Heck GR, Feldmann P (2007). Control of coleopteran insect pests through RNA interference.. Nat Biotech.

[8] Mao YB, Cai WJ, Wang JW, Hong GJ, Tao XY (2007). Silencing a cotton bollworm P450 monooxygenase gene by plant-mediated RNAi impairs larval tolerance of gossypol.. Nat Biotech.

[9] Kamath RS, Martinez-Campos M, Zipperlen P, Fraser AG, Ahringer J (2000). Effectiveness of specific RNA-mediated interference through ingested double-stranded RNA in *Caenorhabditis elegans*.. Genome Biol.

[10] Turner CT, Davy MW, MacDiarmid RM, Plummer KM, Birch NP (2006). RNA interference in the light brown apple moth, *Epiphyas postvittana* (Walker) induced by double-stranded RNA feeding.. Insect Mol Biol.

[11] Meyering-Vos M, Muller A (2007). RNA interference suggests sulfakinins as satiety effectors in the cricket *Gryllus bimaculatus*.. J Insect Physiol.

[12] Zhou X, Wheeler MM, Oi FM, Scharf ME (2008). RNA interference in the termite *Reticulitermes flavipes* through ingestion of double-stranded RNA.. Insect Biochem Mol Biol.

[13] Timmons L, Fire A (1998). Specific interference by ingested dsRNA.. Nature.

[14] Newmark PA, Reddien PW, Cebria F, Alvarado AS (2003). Ingestion of bacterially expressed double-stranded RNA inhibits gene expression in planarians.. Proc Natl Acad Sci USA.

[15] Fraser AG, Kamath RS, Zipperlen P, Martinez-Campos M, Sohrmann M (2000). Functional genomic analysis of *C. elegans* chromosome I by systematic RNA interference.. Nature.

[16] Merzendorfer H (2006). Insect chitin synthases: a review.. J Comp Physiol B: Biochemical, Systemic, and Environmental Physiology.

[17] Chen X, Yang X, Senthil Kumar N, Tang B, Sun X (2007). The class A chitin synthase gene of *Spodoptera exigua*: molecular cloning and expression patterns.. Insect Biochem Mol Biol.

[18] Merzendorfer H, Zimoch L (2003). Chitin metabolism in insects: structure, function and regulation of chitin synthases and chitinases.. J Exp Biol.

[19] Zimoch L, Hogenkamp DG, Kramer KJ, Muthukrishnan S, Merzendorfer H (2005). Regulation of chitin synthesis in the larval midgut of *Manduca sexta*.. Insect Biochem Mol Biol.

[20] Chen X, Tian H, Zou L, Tang B, Hu J (2008). Disruption of *Spodoptera exigua* larval development by silencing chitin synthase gene A with RNA interference.. B Entomol Res.

[21] Winston WM, Molodowitch C, Hunter CP (2002). Systemic RNAi in *C. elegans* requires the putative transmembrane protein SID-1.. Science.

[22] Gura T (2000). A silence that speaks volumes.. Nature.

[23] Retnakaran Arthur, Wright JamesE, Wright JamesE (1987). Control of insect pests with benzoylphenyl ureas.. Chitin and Benzoylphenyl Ureas.

[24] Rajagopal R, Sivakumar S, Agrawal N, Malhotra P, Bhatnagar RK (2002). Silencing of midgut Aminopeptidase N of *Spodoptera litura* by double-stranded RNA establishes its role as *Bacillus thuringiensis* toxin receptor.. J Biol Chem.

[25] Araujo RN, Santos A, Pinto FS, Gontijo NF, Lehane MJ (2006). RNA interference of the salivary gland nitrophorin 2 in the triatomine bug *Rhodnius prolixus* (Hemiptera: Reduviidae) by dsRNA ingestion or injection.. Insect Biochem Mol Biol.

[26] Tenllado F, Martinez-Garcia B, Vargas M, az-Ruiz J (2003). Crude extracts of bacterially expressed dsRNA can be used to protect plants against virus infections.. BMC Biotech.

[27] Arakane Y, Muthukrishnan S, Kramer KJ, Specht CA, Tomoyasu Y (2005). The *Tribolium* chitin synthase genes *TcCHS1* and *TcCHS2* are specialized for synthesis of epidermal cuticle and midgut peritrophic matrix.. Insect Mol Biol.

[28] Tomoyasu Y, Denell RE (2004). Larval RNAi in *Tribolium* (Coleoptera) for analyzing adult development.. Dev Genes Evol.

[29] Sivakumar S, Rajagopal R, Venkatesh GR, Srivastava A, Bhatnagar RK (2007). Knockdown of Aminopeptidase-N from *Helicoverpa armigera* larvae and in transfected Sf21 cells by RNA interference reveals its functional interaction with *Bacillus thuringiensis* Insecticidal Protein Cry1Ac.. J Biol Chem.

[30] Eleftherianos I, Millichap PJ, ffrench-Constant RH, Reynolds SE (2006). RNAi suppression of recognition protein mediated immune responses in the tobacco hornworm *Manduca sexta* causes increased susceptibility to the insect pathogen *Photorhabdus*.. Dev Comp Immunol.

[31] Huang J, Zhang Y, Li M, Wang S, Liu W (2007). RNA interference-mediated silencing of the bursicon gene induces defects in wing expansion of silkworm.. FEBS Letters.

[32] Mito T, Kobayashi C, Sarashina I, Zhang H, Shinahara W (2007). even-skipped has gap-like, pair-rule-like, and segmental functions in the cricket *Gryllus bimaculatus*, a basal, intermediate germ insect (Orthoptera).. Dev Biol.

[33] Ronco M, Uda T, Mito T, Minelli A, Noji S (2008). Antenna and all gnathal appendages are similarly transformed by homothorax knock-down in the cricket *Gryllus bimaculatus*.. Dev Biol.

[34] Kato N, Mueller CR, Fuchs JF, Wessely V, Lan Q (2006). Regulatory mechanisms of chitin biosynthesis and roles of chitin in peritrophic matrix formation in the midgut of adult *Aedes aegypti*.. Insect Biochem Mol Biol.

[35] Cruz J, Mane-Padros D, Belles X, Martin D (2006). Functions of the ecdysone receptor isoform-A in the hemimetabolous insect *Blattella germanica* revealed by systemic RNAi in vivo.. Dev Biol.

[36] Cruz J, Martin D, Belles X (2007). Redundant ecdysis regulatory functions of three nuclear receptor HR3 isoforms in the direct-developing insect *Blattella germanica*.. Mech Dev.

[37] Li G, Pang Y, Chen Q, Su Z, Wen X (2002). Studies on the artificial diet for beet armyworm, *Spodoptera exigua*.. Chinese J Biol Control.

[38] Tamura K, Dudley J, Nei M, Kumar S (2007). MEGA4: Molecular Evolutionary Genetics Analysis (MEGA) software version 4.0.. Mol Biol Evol.

