# Supplementary figures and images for: Developmental Control of a Lepidopteran Pest Spodoptera exigua by Ingestion of Bacteria Expressing dsRNA of a Non-Midgut Gene
Source: PLoS One. 2009 Jul 13;4(7):e6225. doi: 10.1371/journal.pone.0006225 (PMC2704864; doi:10.1371/journal.pone.0006225)

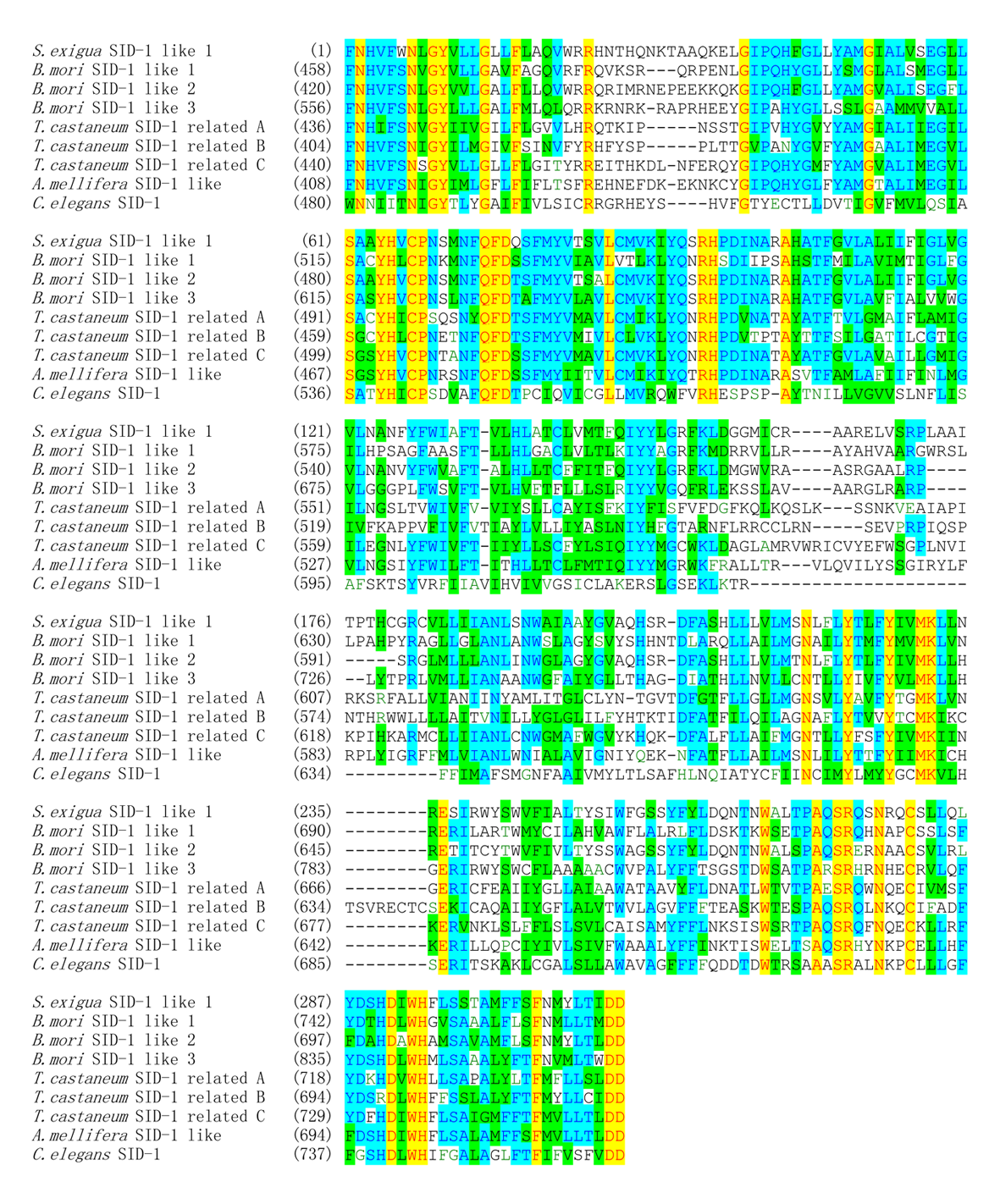

Supplement: Figure S1 — Multiple alignment of Spodoptera exigua SID-1 and other insects or nematode Caenorhabditis elegans SID-1 translated amino acid sequences. S. exigua SID-1 like 1, GenBank accession no. ACM47363; B. mori SID-1 like 1, BAF95805; B. mori SID-1 like 2, BAF95807; B. mori SID-1 like 3, BAF95806; T. castaneum SID-1 related A, NP_001099012; T. castaneum SID-1 related B, NP_001103253; T. castaneum SID-1 related C, NP_001099128; A. mellifera SID-1 like, XP_395167; and C. elegans SID-1, AAL78657. (1.21 MB TIF) [file pone.0006225.s001.tif]

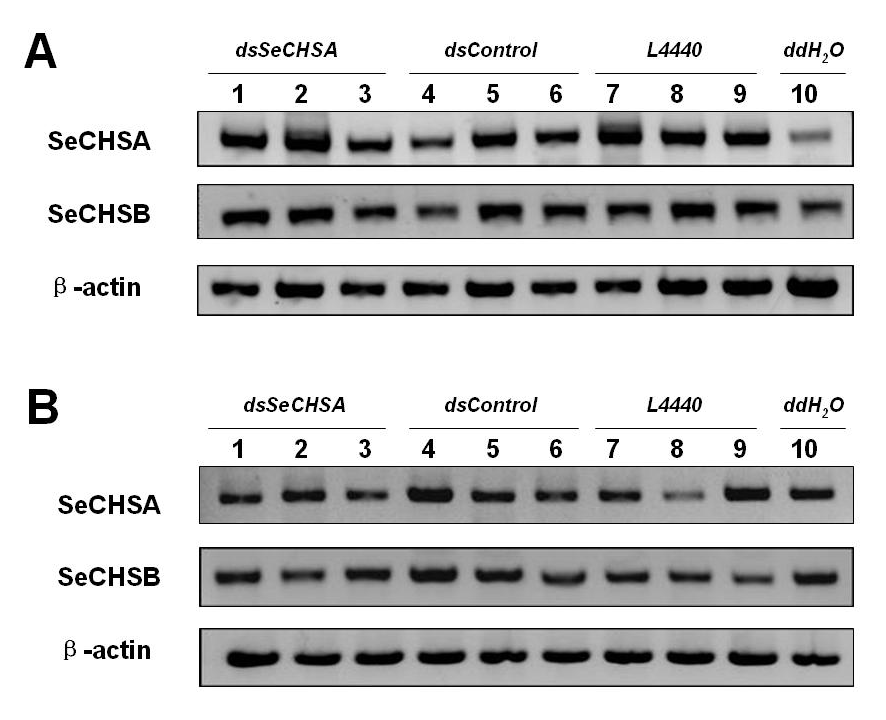

Supplement: Figure S2 — Effects of ingestion of bacteria-expressed dsRNA on transcription of SeCHSA and SeCHSB on day 3 (A) and day 5 (B) post-feeding. Total RNA was extracted from individual larvae feeding on the diet containing bacteria-expressed dsRNA of dsSeCHSA and SeCHSA and SeCHSB transcripts were detected using RT-PCR. RNA from larvae fed on the diet containing the control bacteria with DmWhite (dsControl), L4440 or with ddH2O served as controls. The lane 1, 4 and 7 represent the high bacteria concentration (250×) feeding larvae,the lane 2, 5 and 8 represent the middle bacteria concentration (50×) feeding larvae, and the lane 3, 6, 9 represent the low bacteria concentration (10×) feeding larvae. The house keeping gene β-actin was used as a reference. (0.25 MB TIF) [file pone.0006225.s002.tif]

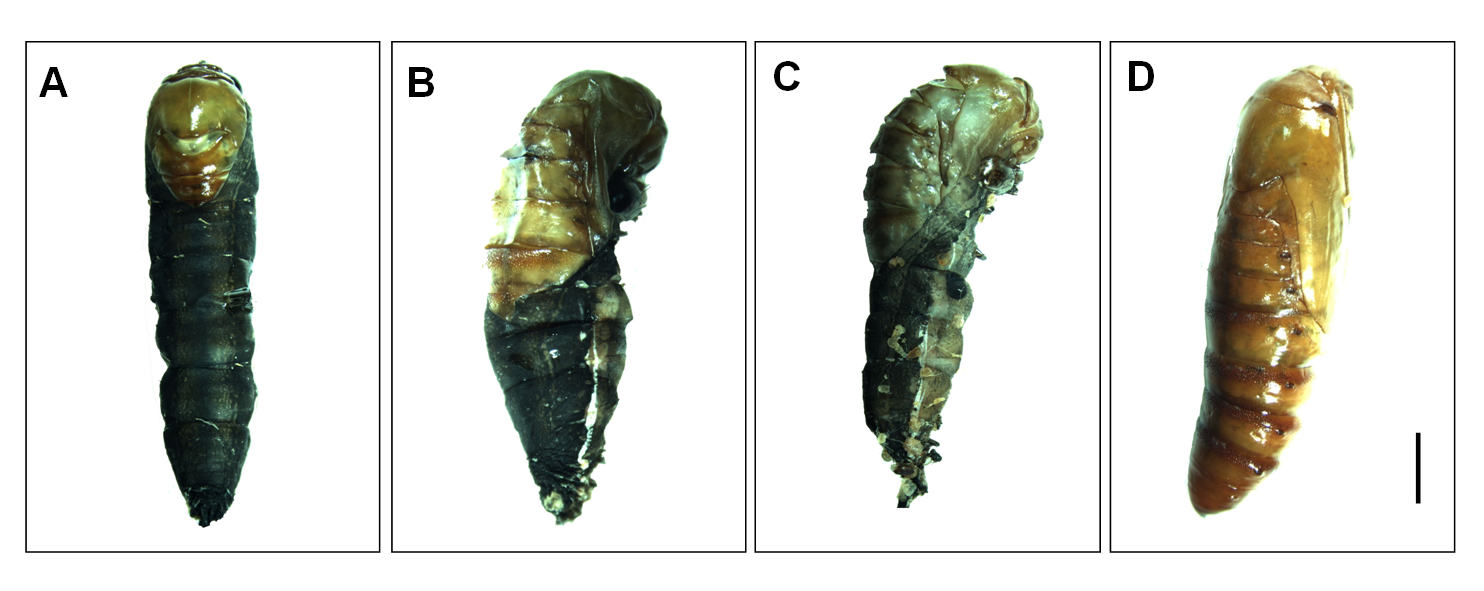

Supplement: Figure S3 — The variable malformed pupae after ingestion of high dose dsRNA in Spodoptera exigua. The frenquency of these phenotypes were nearly the same between dsRNA feeding and controls. A, B and C show the variable phenotypes of pupae that appeared in each group. D shows the normal pupae. The scale bar represents 2 mm. (0.56 MB TIF) [file pone.0006225.s003.tif]

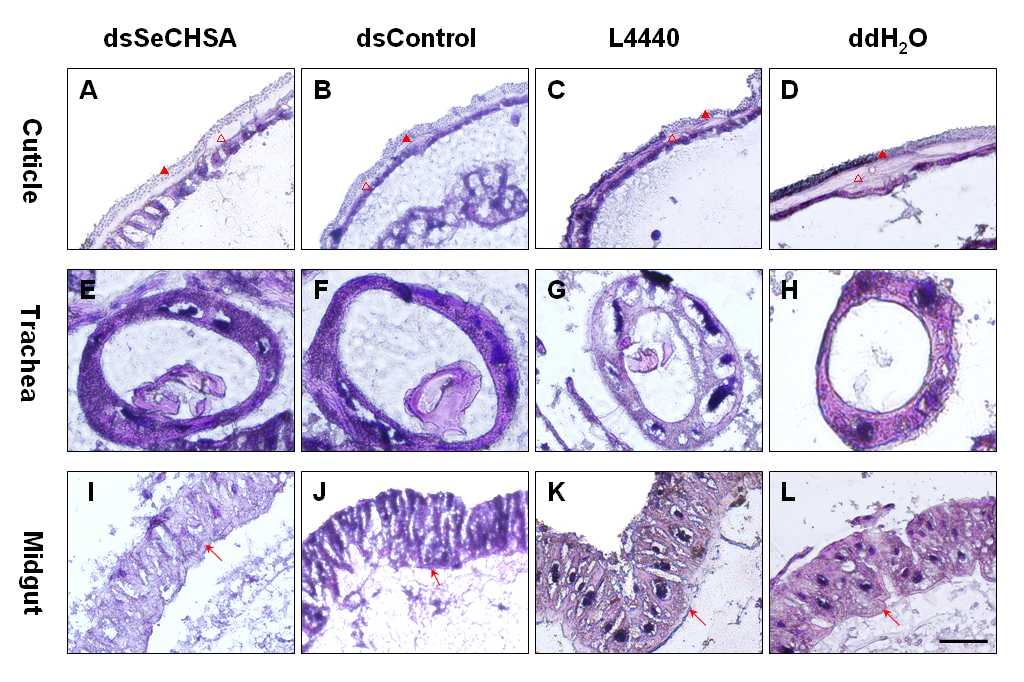

Supplement: Figure S4 — The Effects of ingestion of bacterially-expressed dsRNA (the medium bacteria concentration, 50×) on the cuticle, trachea and midgut peritrophic matrix of Spodoptera exigua larvae. Cuticle, trachea and midgut peritrophic matrix were dissected from the abnormal larvae 9 days after feeding on the diet containing bacterially-expressed SeCHSA dsRNA,or from the larvae at the same stage fed on bacterially-expressed DmWhite dsRNA (Control), control L4440 bacteria or ddH2O. All tissues were stained with Hemotoxylin and eosin (H & E). The filled and open red triangles in A, B, C and D represent exocuticle and endocuticle, respectively; the red arrows in I, J, K and L represent peritrophic matrix of the midgut. Scale bar represents 50 µm. (1.20 MB TIF) [file pone.0006225.s004.tif]

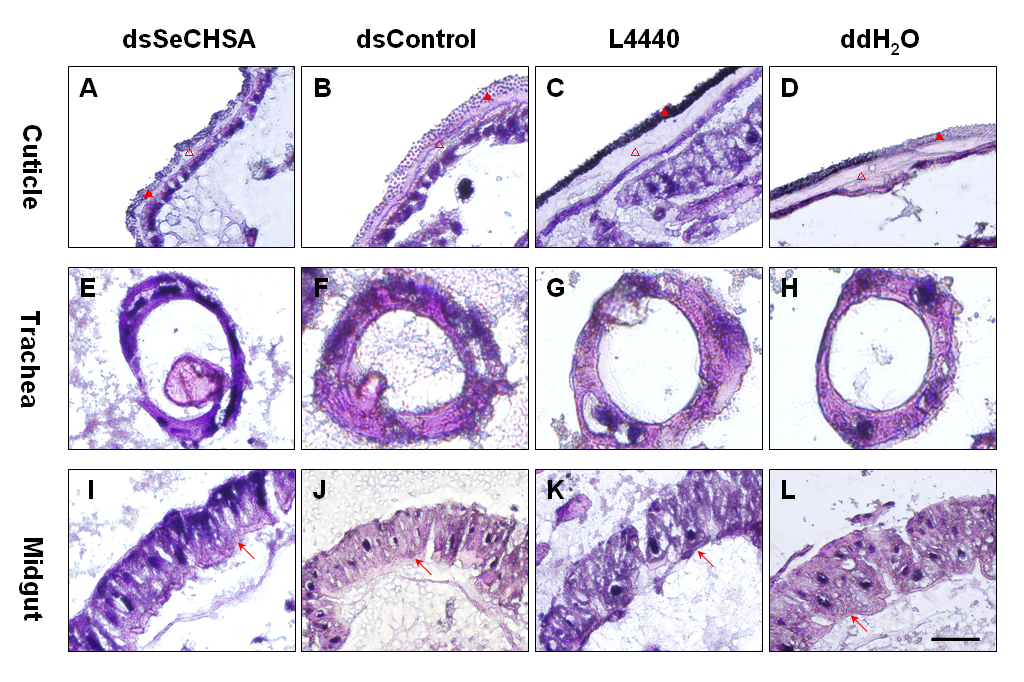

Supplement: Figure S5 — The Effects of ingestion of bacterially-expressed dsRNA (the low bacteria concentration, 10×) on the cuticle, trachea and midgut peritrophic matrix of Spodoptera exigua larvae. Cuticle, trachea and midgut peritrophic matrix were dissected from the abnormal larvae 9 days after feeding on the diet containing bacterially-expressed SeCHSA dsRNA,or from the larvae at the same stage fed on bacterially-expressed DmWhite dsRNA (Control), control L4440 bacteria or ddH2O. All tissues were stained with Hemotoxylin and eosin (H & E). The filled and open red triangles in A, B, C and D represent exocuticle and endocuticle, respectively; the red arrows in I, J, K and L represent peritrophic matrix of the midgut. Scale bar represents 50 µm. (1.19 MB TIF) [file pone.0006225.s005.tif]
